# The roles of the kynurenine pathway in COVID-19 neuropathogenesis

**DOI:** 10.1007/s15010-024-02293-y

**Published:** 2024-05-27

**Authors:** Mona Dehhaghi, Mostafa Heydari, Hamed Kazemi Shariat Panahi, Sharon R. Lewin, Benjamin Heng, Bruce J. Brew, Gilles J. Guillemin

**Affiliations:** 1 Macquarie Medical School, Faculty of Medicine, Health and Human Sciences, Macquarie University, Sydney, NSW Australia; 2 Department of Pharmaceutical Nanotechnology, Faculty of Pharmacy, Tehran University of Medical Science, Tehran, Iran; 3 Department of Infectious Diseases, The University of Melbourne at the Peter Doherty Institute for Infection and Immunity, Melbourne, VIC Australia; 4 Victorian Infectious Diseases Service, The Royal Melbourne Hospital at the Peter Doherty Institute for Infection and Immunity, Melbourne, VIC Australia; 5 Department of Infectious Diseases, The Alfred Hospital and Monash University, Melbourne, VIC Australia; 6 Peter Duncan Neurosciences Unit, St. Vincent’s Centre for Applied Medical Research, Sydney, NSW Australia; 7 Faculty of Medicine and Health, School of Clinical Medicine, UNSW Sydney, NSW Australia; 8 Departments of Neurology and Immunology, St. Vincent’s Hospital, Sydney, NSW Australia; 9 University of Notre Dame, Darlinghurst, Sydney, NSW Australia; 10 Department of Chemistry, Faculty of Mathematics and Natural Sciences, Institut Pertanian Bogor University, Bogor, Indonesia

**Keywords:** SARS-CoV-2, COVID-19, Tryptophan, Kynurenine pathway, Long COVID, Neurological manifestations

## Abstract

The severe acute respiratory syndrome coronavirus 2 (SARS-CoV-2) is the causative agent of the highly contagious respiratory disease Corona Virus Disease 2019 (COVID-19) that may lead to various neurological and psychological disorders that can be acute, lasting days to weeks or months and possibly longer. The latter is known as long-COVID or more recently post-acute sequelae of COVID (PASC). During acute COVID-19 infection, a strong inflammatory response, known as the cytokine storm, occurs in some patients. The levels of interferon‐γ (IFN‐γ), interferon-β (IFN-β), interleukin-6 (IL-6) and tumour necrosis factor-alpha (TNF-α) are particularly increased. These cytokines are known to activate the enzyme indoleamine 2,3-dioxygenase 1 (IDO-1), catalysing the first step of tryptophan (Trp) catabolism through the kynurenine pathway (KP) leading to the production of several neurotoxic and immunosuppressive metabolites. There is already data showing elevation in KP metabolites both acutely and in PASC, especially regarding cognitive impairment. Thus, it is likely that KP involvement is significant in SARS-CoV-2 pathogenesis especially neurologically.

## Introduction

Coronaviruses are characterized as a diverse group of infectious viruses in animals and humans [1, 2]. Two coronaviruses, namely severe acute respiratory syndrome coronavirus (SARS-CoV) and Middle East respiratory syndrome coronavirus (MERS-CoV), were reported in 2002 and 2012, respectively [3, 4]. Both viruses are considered to be zoonotic pathogens that have been transmitted from animals, possibly from bats to humans [5]. They are the causative agents of fatal respiratory disorders, introducing coronaviruses as a pandemic threat. The highly contagious respiratory disease, *i.e.,* Coronavirus disease 2019 (COVID-19), is caused by a recent mutated form called severe acute respiratory syndrome coronavirus 2 (SARS-CoV-2). The first known infection was reported in December 2019 in the city of Wuhan in China and rapidly spread worldwide and became a global viral pandemic. The mortality of COVID-19 appears to be mainly associated with acute respiratory distress syndrome (ARDS), followed by multi-organ failure [6, 7]. Other serious respiratory, hepatic, cardiovascular and neurological disorders can be observed [8–10]. The World Health Organization (WHO) frequently updates strategies concerning the global management of COVID-19. Despite huge global efforts fighting COVID-19, the pathogenesis and treatment of this disease are yet to be fully understood and much research is focused on identifying key pathways involved in SARS-CoV-2 infection. One promising research approach is the investigation of the kynurenine pathway (KP) in patients suffering from COVID-19. Recently, significant increases in the levels of some KP metabolites such as quinolinic acid (QUIN) and 3- hydroxykynurenine (3-HK) have been found in SARS-CoV-2 ( + ) patients [11]. Importantly, both metabolites have been identified as neurotoxic [12–14]. Moreover, untargeted metabolomics analyses revealed that tryptophan (Trp) metabolism is one of the most significantly modulated pathways affected by SARS-CoV-2 infection [15]. These authors reported marked decreases in Trp and serotonin levels and increases in kynurenine (KYN), picolinic acid (PIC), and kynurenic acid (KYNA), highlighting KP activation in SARS-CoV-2 ( + ) patients [15]. Furthermore, recent results of untargeted metabolite and cytokine data showed that cognitive impairment in post-acute sequelae of COVID (PASC) was associated with KP activation most especially QUIN, 3-hydroxyanthranilic acid (3-HAA), and KYN (p < 0.001); no other variables related to cognitive impairment [16]. Further, sustained activation of the KP for 2 to 8 months (p < 0.0001) was associated with interferon-β (INF-β) in cases with PASC. The present review discusses the mechanisms behind the KP alterations in COVID-19 acute infection and scrutinizes the potential impact of the KP on the incidence of other health implications associated with long COVID.

## The kynurenine pathway: overview and enzymatic regulation

**Fig. 1:**
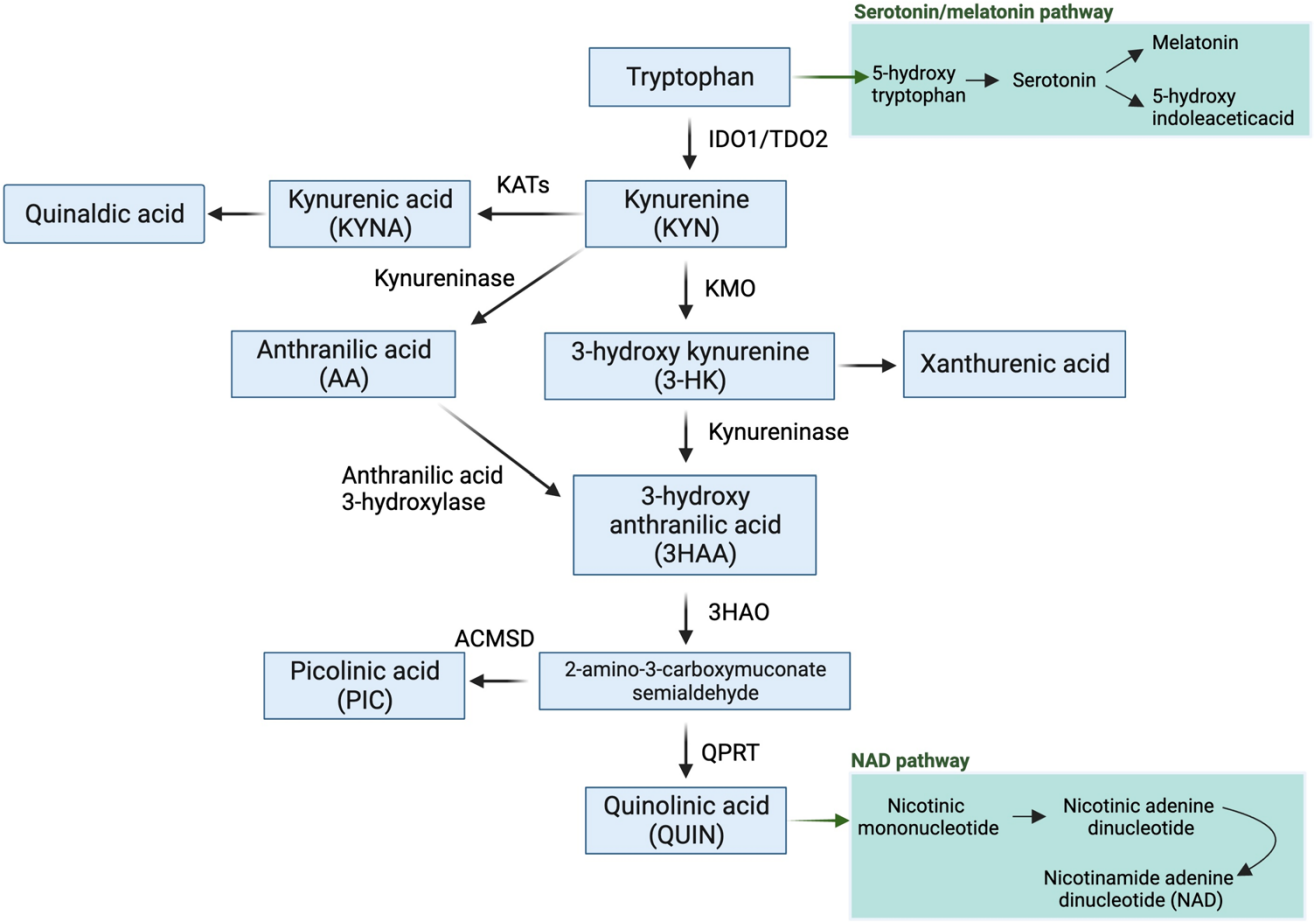
The kynurenine pathway (*KP*) and its derivative pathways. *IDO*-1: Indoleamine-pyrrole 2,3-dioxygenase; *TDO*2: Tryptophan 2,3-dioxygenase; *KATs*: Kynurenine aminotransferase; *KMO*: Kynurenine 3-monooxygenase; *HAAO*: 3-hydroxyanthranilate 3,4-dioxygenase; *QPRT*: Quinolinate phosphoribosyl transferase; *ACMSD*: Aminocarboxymuconate-semialdehyde decarboxylase. Created with biorender.com

l-Tryptophan is an essential amino acid, metabolized through the KP and serotonin pathway [17–20]. Approximately 95% of available Trp is catabolised through the KP into several neuroactive metabolites (*i.e.* KYNA, KYN, 3-HK and QUIN) and the essential metabolic co-factor nicotinamide adenine dinucleotide (NAD + ) (Fig. 1) [19, 20]. KP metabolites are synthesised in various human tissues, however, different human cell types produce different KP metabolites. For example, neuroprotective KYNA is produced mainly by astrocytes, whereas the excitotoxin QUIN is mainly produced by activated monocyte lineage cells [14, 21]. Indoleamine 2,3-dioxygenase 1 (IDO-1) catalyses the first step of the KP, converting Trp into KYN through the intermediate *N*-formylkynurenine. Further, KYN is converted to 3-HK, 3-HAA, QUIN, and ultimately NAD + .KYN can also be converted into KYNA by kynurenine aminotransferase (KAT) enzymes [22]. KYNA, a neuroprotective metabolite, is an endogenous *N*-methyl-d-aspartate (NMDA) receptor antagonist. It also acts as a negative allosteric modulator of α7 nicotinic acetylcholine receptor (α7nAChR) [22, 23] and has an important role in the regulation of the immune system through its interaction with aryl hydrocarbon receptor (AhR) [24]. QUIN acts mainly as an NMDA receptor agonist that induces excitotoxicity in neurons. However, QUIN has several other mechanisms of toxicity through increasing reactive oxygen species (ROS) and reactive nitrogen species (RNS) formation, enhancing glutamate release by neurons and inhibiting its uptake by astrocytes, lipid peroxidation, energy depletion, and increasing production of nitric oxide [25–28] (Fig. 1).

**Fig. 2:**
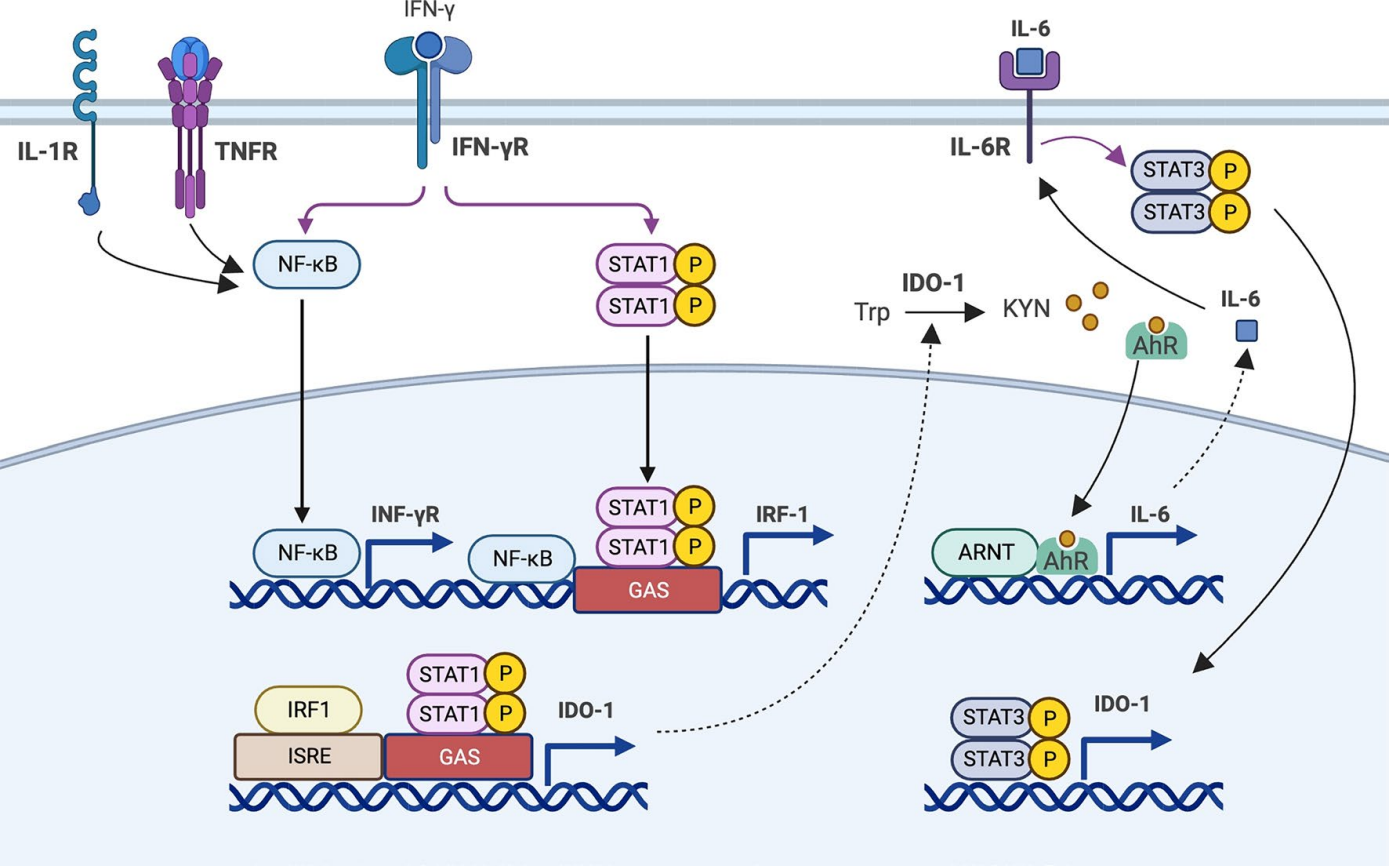
Regulation of IDO-1 transcription by the immune system. IFN-γ induces the expression of IDO-1 through STAT-1 and NF-κB. Binding IFN-γ to IFN-γR initiates phosphorylation of STAT-1 and binding to the GAS sequence. STAT-1 and NF-κB promote IRF1 synthesis, which binds to the ISRE sites in IDO1 and enhances IDO-1 transcription. In addition to IFN-γ, proinflammatory cytokines such as IL-1 and TNFα induce the expression of IDO-1 through NF-κB. IDo-1 activation follows by conversion of Trp to KYN. Activation of AhR by KYN induces the production of IL-6 and sustains IDO-1 expression via AhR–IL-6–STAT3 positive feedback loop. Created with biorender.com

IDO-1 has an evolutionary paralog and a functional ortholog called IDO-2 and trp-2,3-dioxygenase (TDO), respectively. These enzymes have similar functions in the first step of the KP; however, IDO-1 is highly expressed by microglia, macrophages and neuronal cells [19, 21]. IDO-1 expression occurs in the placenta, mucosa of the gut, certain tumours, tumour-draining lymph nodes, and immune cells. The proinflammatory cytokine interferon-γ (IFN-γ) is a highly potent inducer of IDO-1 expression in several cell types such as macrophages, fibroblasts, and dendritic cells (DCs) [29]. IDO-1 expression induced by IFN-γ can be enhanced through the involvement of other proinflammatory cytokines such as interleukin-1β (IL-1β) and tumour necrosis factor α (TNFα) [30, 31]. Both IL-1β and TNF-α increase the expression of IFN-γ receptors (IFN-γR) through the nuclear factor κB (NF-κB). IFN-γ regulates *IDO-1* transcription via transducer and activator of transcription 1 (STAT-1) and NF-κB. Upon binding of IFN-γ to its receptor, phosphorylation of STAT-1 occurs leading to its dimerization and binding to the regulatory gamma activation sequence (GAS1) located upstream of the *IDO-1*. In addition, IFN-γ-regulated factor 1 (IRF1) is transcribed in a NF-κB-STAT-1 dependent manner, which subsequently regulates the *IDO-1* transcription through binding to the IFN-stimulated response element (ISRE) [32, 33]. Activation of AhR by KYN stimulates the production of interleukin-6 (IL-6) and sustains IDO-1 expression via AhR–IL-6–STAT3 positive feedback loop (Fig. 2). Expression of *IDO-1* induced by IFN-γ could be inhibited by interleukin-4 (IL-4). The study conducted by Musso et al. [34] elucidated that IL-4 not only prevents the induction of IDO mRNA by IFN-γ but also substantially reduces IDO enzymatic activity, marking the first evidence of cytokine-mediated negative regulation of IDO expression [34]. However, despite these findings, the precise mechanisms underlying the inhibition of IDO-1 by IL-4 remain largely unexplored and poorly understood. The complexities involved in the cytokine signalling pathways and the modulation of IDO-1 activity suggest that there are significant gaps in our understanding of the interaction between IL-4 and IDO-1 expression. Like IDO-1, TDO catabolises Trp to KYN, however, its regulation pathways are different. Regulation of TDO can occur through hormonal induction by glucocorticoids, allosteric inhibition by nicotinamide adenine dinucleotide phosphate (NAD(P)H), and substrate activation [35–37].

Apart from cytokines, the gut microbiome has also the capacity to modulate IDO-1 [38–40]. Notably, among the metabolites derived from gut bacteria, short-chain fatty acids (SCFAs) including butyrate, propionate, and acetate play vital roles in host metabolism and immune development [41]. It has been reported that expression of intestinal epithelial IDO-1 could be regulated by butyrate through the two different mechanisms. Initially, butyrate decreases the expression of STAT1, resulting in the suppression of IFN-γ-inducing transcription of IDO-1. Moreover, an additional mechanism has been suggested by which butyrate decreases the transcription of IDO-1 independently of STAT1. This occurrence is linked to its function as a histone deacetylase (HDAC) inhibitor [38] (Fig. 2).

Similar to IDO-1, other KP enzymes may be regulated by the proinflammatory cytokines. Kynurenine 3-monooxygenase (KMO) which converts KYN to 3-HK can be induced by IFN-γ and IL-1β [42, 43]. Both IFN-γ and IL-1β induce the expression of the *Kmo* gene as well as its upstream enzymes (*i.e.,* IDO-1, TDO) resulting in increased availability of KYN as KMO substrate and subsequently elevated levels of 3-HK. Both pharmacological inhibition of KMO and knockout of the *Kmo* gene cause increased levels of KYN and its conversion to KYNA [44–46], the latter being performed by kynurenine aminotransferases (KATs) through the KYNA branch (Fig. 1). Various isotypes of KATs (KAT I, II, III, and IV) are found in different tissues and species, which are responsible for metabolization of KYN to KYNA as well as 3-HK to xanthurenic acid (XA). KAT II is the predominant type in the brain of humans with high *K*_m_ values for both KYN and 3-HK (4.7 and 3.8 mM, respectively). Microarray analyses of human carotid atherosclerotic plaques have revealed a positive correlation between the expression of IDO-1, TDO, and KMO and pro-inflammatory and metalloprotease genes. Conversely, the expression of KAT1 and KAT2 showed a negative correlation with pro-inflammatory molecules [47]. Moreover, IL-4 has been shown to have regulatory effects on KAT II. IL-4 is mainly associated with responses of effector Th2 cells [48], however, its inhibitory effect on KAT II leads to a reduction in extracellular levels of KYNA [49]. Inhibiting KAT II has been associated with restoring nicotine-evoked glutamatergic activity in the cortex of rats, suggesting a potential role in addressing cognitive functions [50]. In addition, KAT II inhibitors have been explored for their potential in pro-cognitive interventions in conditions such as schizophrenia and other major brain diseases [51].

## Interplay between the kynurenine pathway and immune regulation

KP metabolites play important roles in the regulation of both innate and adaptive immune responses. Expression of IDO-1 occurs in several types of immune cells such as regulatory B cells, DCs, monocytes, and macrophages [52–54]. Activation of IDO-1 in these cells affects the immune responses by generating the immunoregulatory metabolites, especially KYN. IFN-γ induces IDO-1 expression in antigen-presenting cells (APCs) such as macrophages, monocytes, and DCs followed by Trp degradation and the production of KP metabolites [55, 56]. DCs are a group of professional APCs, playing critical regulatory roles in immune responses [57]. These cells are able to produce several cytokines such as TNF-α, IL-6, interlukin-12 (IL-12), interlukin-23 (IL-23), and type I and III of interferons [58, 59]. The ability of plasmacytoid DCs in the production of type I interferons emphasizes their key roles in viral infections. Conventional DCs are known as the important APCs in the innate immune system which present the antigens to the naive T lymphocytes, inducing their proliferation and differentiation to regulatory T cells (Treg) [60, 61]. In addition to the inflammatory roles of DCs, these cells can express high levels of IDO-1 and induce immunotolerance. Upregulation of IDO-1 is followed by KYN production and activation of the AhR in DCs. This condition induces the tolerogenic phenotype of DCs, in which they downregulate the expression of CD80 and CD40 [62]. Moreover, IDO-1-expressing DCs increase the expression of transforming growth factor beta (TGFβ)-mediated FoxP3 in naïve CD4+ T cells, leading to the differentiation of CD4+ T cells to Treg cells [63–65]. Simultaneously, suppressing IL-6 production in CD4+ T cells inhibits the transformation of these cells to Th17-like effector T-cells [66]. IFN-γ-induced IDO-1 expression also occurs in monocytes and macrophages. IDO-1 induction in monocytes mediates the transformation of monocytes into M2-type macrophages, which is associated with the production of IL-4 and interlukin-10 (IL-10) as well as immunomodulatory functions. In vitro studies have reported that treatment of monocytes with IFN-γ significantly increased the ratio of M2/M1. Conversely, silencing IDO-1 induced differentiation of monocytes to pro-inflammatory M1-type macrophages [67].

## The kynurenine pathway and COVID-19: metabolic disturbances and consequences

The increased levels of pro-inflammatory cytokines in acute COVID-19 refer to the cytokine storm [68, 69]. As mentioned earlier, IFN-γ is the most important pro-inflammatory cytokine to induce IDO-1, leading to increased Trp degradation through the KP. Other pro-inflammatory cytokines such as TNF‐α and IL-1β can also induce the expression of IDO-1 through the IFN-γ. IL-6 can also trigger the expression of IDO-1 through the AhR-STAT3-IL-6 loop (Fig. 2). Increased Trp metabolism and induction of IDO-1 in acute COVID-19 cases have been reported in several studies (Table 1). A metabolomic study on 33 patients with COVID-19 reported significant alterations in Trp metabolism in association with IL-6 in COVID-19 patients. It is important to highlight that their findings indicated that Trp metabolism was the pathway most dysregulated during acute COVID-19 infection [15]. this study showed increased levels of KYN, KYNA, and PIC as well as decreased levels of Trp, indole pyruvate and serotonin in acute COVID-19 cases [15]. Similarly, Fraser et al. reported increased KYN in COVID-19 patients admitted to the intensive care unit (ICU) compared to the healthy controls [70]. Another study by Lionetto et al. assessed the levels of KP metabolites in the serum of three groups including SARS-CoV-2 positive patients, SARS-CoV-2 negative cases, and individuals who were admitted to the emergency department but not for COVID-19 infection. Their findings indicated that the KYN/Trp ratio was significantly higher in SARS-CoV-2 positive patients compared to the two other groups. Importantly, this ratio was the highest in acute COVID-19 patients with severe lymphopenia [71]. Marín-Corral et al. also suggested the association between alterations in KP metabolites and acute COVID-19 severity. They studied ceramide metabolism, the KP, and NAD + availability in 49 hospitalized acute COVID-19 patients with moderate, severe, and critical conditions. Their findings suggested that Trp and KP metabolites correlated with the severity of the disease. Decreased levels of Trp and increased concentrations of KYN and 3-HK were associated with acute COVID-19 severity [72]. In another study, Lawler et al. conducted a metabolic phenotyping study in acute COVID-19 patients. They reported elevated levels of 3-HK, KYN, and QUIN in the plasma of SARS-CoV-2 positive cases which were associated with the severity of the disease [11]. Sex-related differences have also been studied in immune response to acute COVID-19 and levels of KP metabolites. The results of a metabolomics analysis of sera obtained from acute COVID-19 cases conducted by Cai et al. indicated in male patients the levels of Trp, KYNA, and KYNA/KYN ratio were associated with age, inflammation, and disease severity [73]. Moreover, a negative correlation was reported between the levels of soluble CD40L (sCD40L), platelet-derived growth factor (PDGFs), eotaxin, and the number of T cells in cases with high KYNA/KYN ratio, suggesting a decrease in the immune response to acute SARS-CoV-2 in male COVID-19 patients [73]. In another study, Lionetto et al. reported the levels of KYN/Trp in acute COVID-19 male patients were significantly greater than KYN/Trp ratio in females [71] (Table 1).

**Table 1:** Alterations of KP metabolites in acute COVID-19 patients

KP metabolites	Type of sample	Number of patients and controls	Main Findings	References
KYN KYNA PIC, Trp	Serum	SARS-CoV2-positive: 33 SARS-CoV2-negative: 16	-Significantly increased levels of KYN, KYNA, and PIC and decreased levels of Trp in COVID-19 patients -Elevated amounts of KP metabolites were associated with levels of IL-6	[15]
KYN	Plasma	SARS-CoV2-positive: 10 SARS-CoV2-negative: 10	-Significantly increased levels of KYN in COVID-19 patients -Arginine/kynurenine ratio presented as a biomarker for classification of COVID-19 patients and healthy controls	[70]
KYN Trp	Serum	SARS-CoV2-positive: 89 Healthy subjects: 239 SARS-CoV2-negative: 305	-Increased levels of KYN and KYN/Trp ratio and decreased levels of Trp in COVID-19 patients -Increased KYN/Trp ratio was higher in males than females	[71]
3-HK KYN QUIN Trp	Plasma	SARS-CoV2-positive: 10 SARS-CoV2-negative: 49	-Increased levels of 3-HK, KYN, and QUIN Trp in COVID-19 patients -Alterations in the metabolites were positively correlated with levels of pro-inflammatory cytokines	[11]
3-HK KYN Trp	Plasma	COVID-19 cases: Moderate: 13 Severe: 10 Critical: 26	-Increased levels of 3-HK and KYN and decreased level of Trp in COVID-19 patients -Alterations in KP metabolites were associated with disease severity	[72]
KYNA KYN	Serum	SARS-CoV2-positive: 39 Uninfected health care workers: 20	-KYNA/KYN ratio was associated with disease severity -Increased KYNA/KYN was higher in males with severe disease -No significant difference in KYNA/KYN ratio was observed in females	[73]
KYNA QUIN	Serum	SARS-CoV2-positive: 40 SARS-CoV2-negative: 40	-Levels of KYNA higher (2.5-fold) in males with COVID-19 with -Increased levels of QUIN was increased in COVID-19 patients -Both KYNA and QUIN had potential sex-association	[180]
AA 3-HK	Plasma	COVID-19 cases: Mild: 23 Moderate: 21 Critical: 28 Healthy controls: 29	-AA was significantly higher in critical patients in comparison to the moderate and mild cases -Increased levels of 3-HK in COVID-19 patients in correlation with disease severity	[181]
KYN Trp	Plasms	SARS-CoV2-positive: 148 (31 patients died due to severe disease)	-High levels of KYN, and KYN/Trp ratio in severe patients -People who died after 90 days had a higher ratio of KYN/Trp compared to patients who recovered	[182]
KYN	Plasma	SARS-CoV2-positive: 151 SARS-CoV2-negative: 302	-Increased levels of KYN in COVID-19 patients -Elevated levels of KYN in 12 patients with post-COVID symptoms after > 20 weeks	[183]
KYN, KYNA, 3-HK, PIC	Serum	SARS-CoV2-positive: 70 SARS-CoV2-negative: 30	-Increased levels of KYN, KYNA, 3-HK, PIC, and KYN/Trp ratio in COVID-19 patients -Decreased levels of Trp in COVID-19 patients -The higher KYN/Trp ratio and increased levels of PIC were associated with disease severity	[184]

*KYN* Kynurenine, *KYNA* Kynurenic acid,* PIC* Picolinic acid, *Trp* Tryptophan, 3-*HK*: 3-Hydroxykynurenine, *QUIN* Quinolinic acid, *AA* Anthranilic acid

## AhR and IDO-1: key signalling pathways in COVID-19 pathogenesis

Cellular adaptation constantly occurs in response to the alterations to the cellular environment resulting from various factors such as diet, host and microflora metabolisms and environmental changes. Cells use multiple molecules as sensors to detect physiological and pathological stimuli. The AhR is one of these sensors, a ligand-activated transcription factor, that is mainly involved in tissue homeostasis and inflammation processes [74]. The AhR is captured in the cytoplasm by proteins such as protein kinase SRC, p23, heat shock protein 90 (HSP90), and AhR-interacting protein. The binding of ligand to AhR triggers its translocation to the nucleus. Eventually, the AhR forms a heterodimer with the AhR nuclear translocator that can regulate the transcription of target genes by binding to DNA. One of the endogenous AhR ligands that is synthesized by immune cells is KYN [24, 75].

**Fig. 3:**
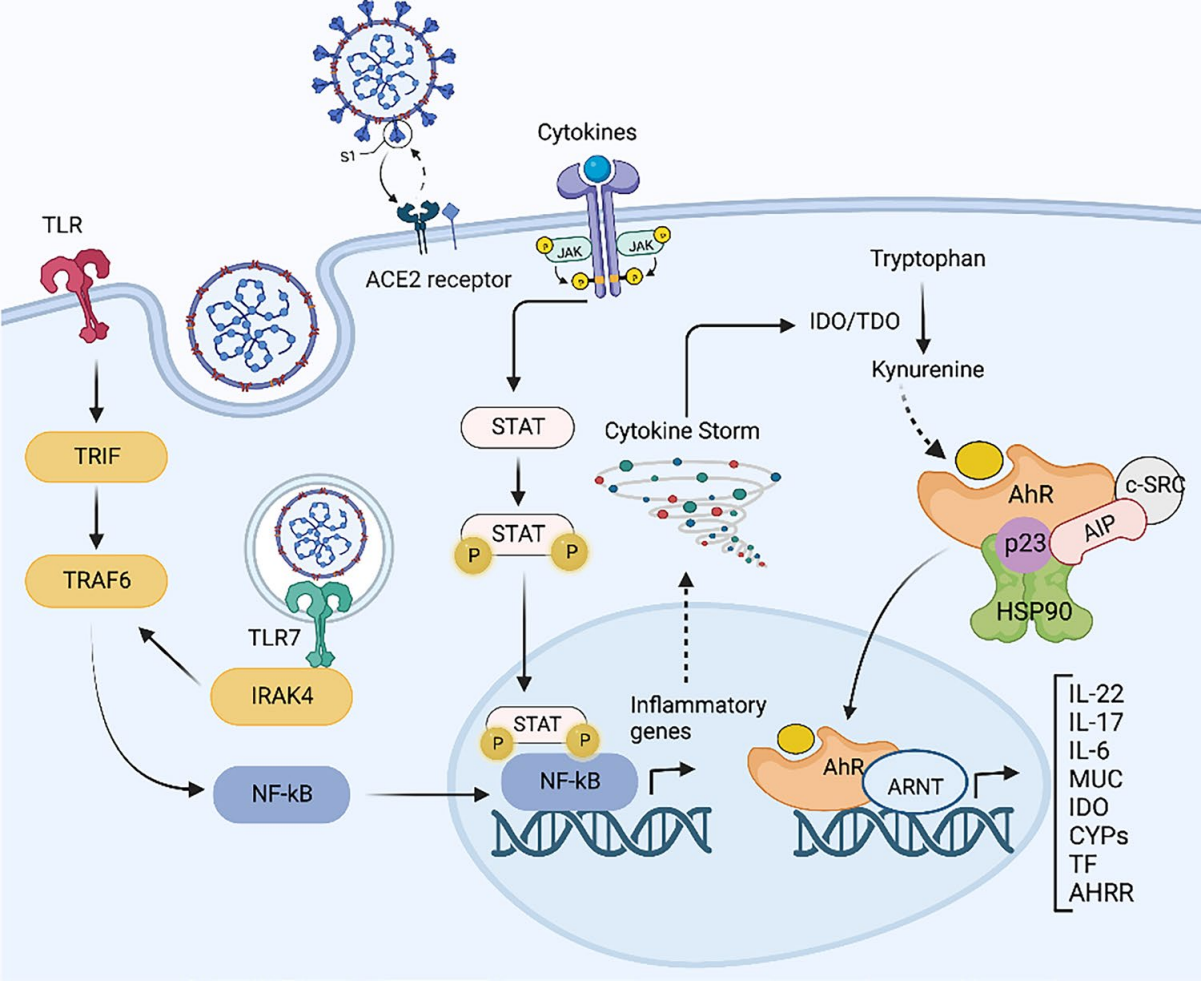
IDO/TDO-AhR signalling pathways during COVID-19 disease. Upon SARS-CoV-2 immune-cell entry, viral RNA is recognized by cell receptors such as Toll-like receptors (TLR) resulting in activation of the NF-ĸB and expression of inflammatory genes. Inflammatory molecules such as IFN-β and IFN-γ activate IDO-1/TDO which catalyse the conversion of Trp to the AhR agonist KYN. AhR is inactive in cytoplasm, which is complexed with HSP90, AIP, p23, and SRC. Upon agonist binding, AhR is translocated to the nucleus where it binds DNA-responsive elements to regulate the expression of numerous genes such as IL-22, IL-17, IL-6, mucins, IDO, CYPs, TF, and AHRR. TLR, Toll-like receptors; NF-ĸB, nuclear factor of кB; ARNT, AHR nuclear translocator; MUC, mucins; CYPs, cytochromes P450; TF, tissue factor; AHRR, AHR repressor. Created with biorender.com

Following binding to the angiotensin-converting enzyme 2 (ACE2) receptor, SARS-CoV-2 enters cells by activating the viral spike glycoproteins via transmembrane serine protease 2 (TMPRSS2) to engage with enzymes expressed by the surfactant secreting alveolar cells of the lung [76]. Toll-like receptors (TLRs) that are key factors in the regulation of innate immune response, play important roles in the recognition of SARS-CoV-2 (Fig. 3). The envelope proteins of SARS-CoV-2 are recognized by TLR-2 [77] and viral RNA (*i.e.,* ssRNA) is recognized by TLR-4, TLR-7 and TLR-8 [78]. Interaction between viral components and TLRs activates NF-κB and promotes the expression of inflammatory cytokines that will induce IDO-1 (Fig. 3). Sustained activation of AhR can also result from the IDO-1-AhR-IDO-1 positive feedback loop. Therefore, a chronic inflammatory state, associated with pathogen persistence, prolongs AhR activation [79, 80]. IDO-1-AhR-IDO-1 loop contributes to Trp depletion through the KP. Upon detection of IDO-1-induced Trp depletion by general control nonderepressible 2 (GCN2) kinase, positive feedback for inducing IDO-1 is initiated, where IL-6 acts as a synergistic molecule. [63]. The KYN-activated AhR induces IL-6 production and promotes IDO-1 expression through the AhR–IL-6–STAT3 positive feedback loop [32, 33].

GCN2 kinase promotes ACE2 expression in CCD 841 cells in response to amino acid deficiency, suggesting a potential role in disease severity [81]. Activation of GCN2 kinase also inhibits the mechanistic target of rapamycin (mTOR) leading to dysregulation of T-cell’s function and apoptosis [63]. Moreover, KYN induces fatty acid β-oxidation by activating AhR in T-cells and promotes immunosuppression. Dysregulation of fatty acid metabolism especially fatty acid oxidation has been shown to increase in COVID-19 cases [82]. In a recent study in mice, it was speculated that coronavirus may upregulate the expression of proviral TCDD-inducible-PARP (TiPARP) and modulate cytokines by activating AhR through an IDO-1-independent manner [83]. Bypassing the IDO-1-KYN-AhR pathway can lead to an unrestricted upregulation of downstream effectors. For example, the proviral factor TiPARP, which modifies the gene expression of the cytokines IL-1β, IL-10 and TNF-α [80, 84] then induces IDO-1, resulting in further over-activation of AhR and leads to an IDO1-AhR-IDO-1 positive feedback loop. COVID-19 could increase the presence of other pathogens and comorbidities that activate AhR via IDO-1-dependent mechanisms [85] (Fig. 3).

## Neurological and psychiatric conditions associated with COVID-19

### Infection of the central nervous system by SARS-CoV-2

The clinical severity of acute COVID-19 can significantly vary between patients. Patients with severe infection may suffer one or more clinical symptoms such as respiratory failure that requires artificial ventilation, multi-organ impairment, and systemic complications (*e.g.,* sepsis, septic shock) [86]. Despite phylogenetic similarity, the affinity of SARS-CoV-1 for the ACE2 is 10- to 20-fold lower than SARS-CoV-2 [87]. Some viruses can invade the central nervous system (CNS) by infecting the olfactory system, peripheral nerves or endothelium (hematogenous route) [88]. The neurological effects observed in some COVID-19 patients may be due to exacerbated immune activation and inflammation within the CNS. For example, Lee et al. reported blood vessel damage and inflammation in the brains of deceased COVID-19 patients without detection of SARS-CoV-2. According to the post-mortem magnetic resonance microscopy and histopathological studies, microvascular injury and leaky blood vessels were found in the patient’s brains. These findings suggested that the presence of activated microglia, macrophages, and CD3 + and CD8 + T cells in the affected regions may contribute to brain vessel damage [89]. So far, only limited COVID-19 nucleic acid or protein particles have been detected in autopsy brain tissue of patients with severe COVID-19 [89, 90]. Stein et al. utilized a multi-faceted approach to detect the presence of the virus in CNS. This investigation implemented a secondary in situ hybridization assay specifically targeting the viral RNA, in conjunction with immunofluorescence and immunohistochemistry-based assays to detect the viral protein in CNS tissues, which had previously tested positive for SARS-CoV-2 via droplet digital PCR (ddPCR). The presence of SARS-CoV-2 RNA was confirmed in the hypothalamus and cerebellum of one case of 11 patients [91]. Crunfli et al. detected SARS-CoV-2 spike proteins in 37% of the cells in the postmortem brain tissues. Notably, a significant portion of these spike-positive cells, accounting for approximately 65.93%, were identified as astrocyte [92].

### Neurological complications of long COVID

**Fig. 4:**
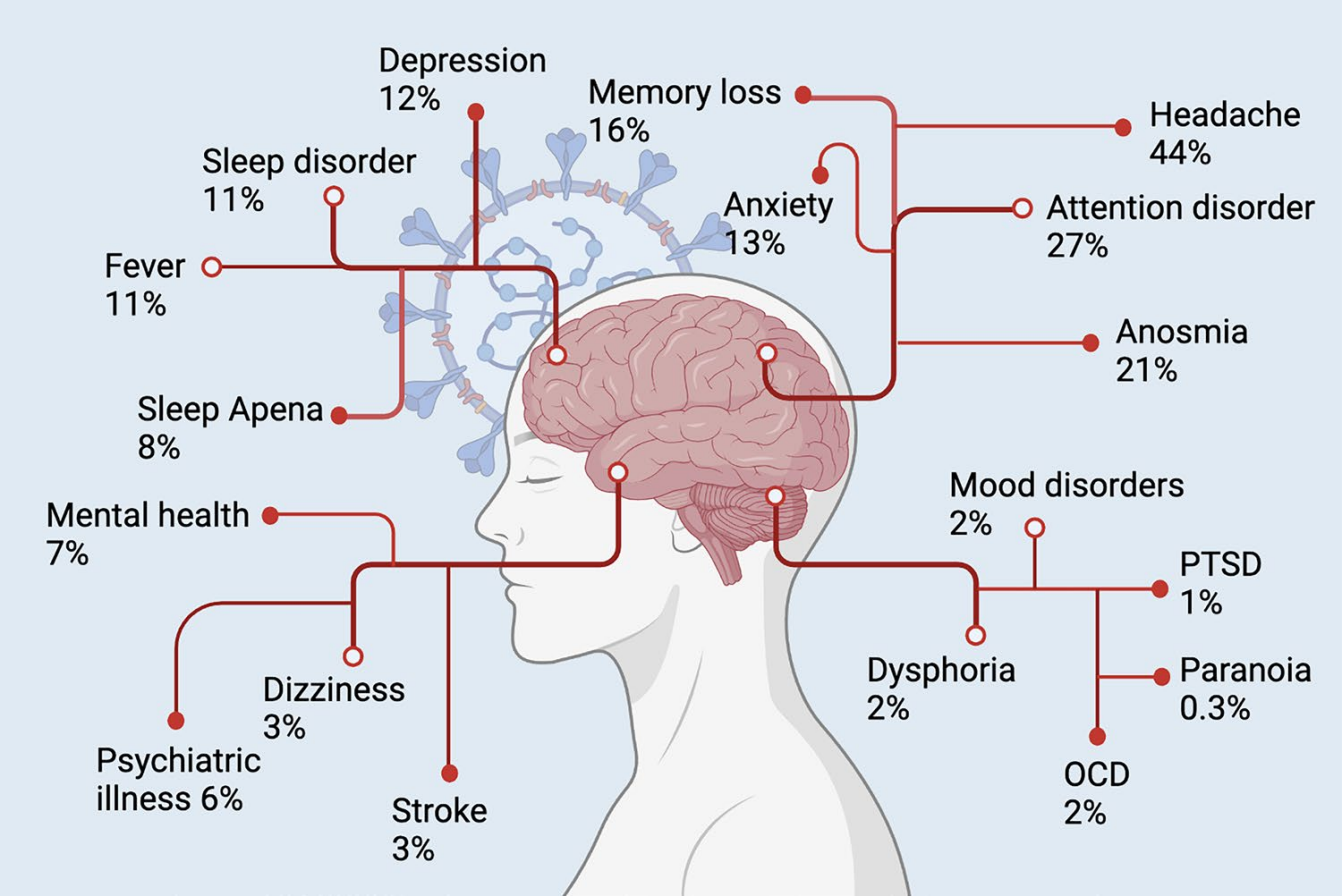
Neuropsychiatric symptoms associated with long-COVID-19. The prevalence of neuropsychiatric symptoms was estimated according to a meta-analysis performed with 15 different peer-reviewed studies including 47,910 patients (age 17–87 years). Adopted from [99]. Created with biorender.com.

According to the WHO, long COVID, also known as a post-COVID condition, is defined as the ongoing or development of new symptoms 3 months after acute COVID which can last at least 2 months [93]. Common symptoms of long COVID include fatigue, cognitive impairment, shortness of breath, and several other health implications that affect everyday functioning [93]. The Centres for Disease Control and Prevention (CDC) also released a definition for long COVID. According to the CDC, some people who were infected with CSRS-CoV-2 may develop symptoms that last for weeks or even months post-acute COVID-19 [94]. It has been reported that six months after recovery from acute COVID-19, up to 57% of patients still showed one or more persistent respiratory-related symptoms and/or fatigue [95]. Importantly, recent findings suggest that females are at much higher risk of development of long COVID symptoms [96–98]. A single-centre cohort study reported that 81.7% of females showed long COVID syndrome, which is a threefold higher risk compared to men [96]. A prospective cohort study conducted by Darley et al. (2021) reported that 21% of long COVID cases developed symptoms consistent with depression [99]. A recent meta-analysis study [100] involving 47,910 patients (aged 17–87 years) reported that more than 80% of individuals experienced at least one long-term effect more than 2 weeks after acute infection. Several neuropsychiatric symptoms, including headache (44%), attention disorder (27%), anosmia (21%), and memory loss (16%), were also reported [100] (Fig. 4). Another meta-analysis study conducted by Lewthwaite et al. reported the application of a treatable traits approach to long COVID [101]. Initially, Lewthwaite et al. identified the most widespread treatable traits associated with long COVID and subsequently examined the existing evidence regarding strategies to address these traits. Through a comprehensive analysis encompassing 22 systematic reviews, they identified 34 symptoms and complications commonly associated with long COVID. According to their study, the most frequent symptom associated with long COVID ( ≥ 12 weeks) was neurological, constituting 25% of complications in the top 10 most prevalent issues across systematic reviews [101]. A meta-analysis of 120,970 COVID-19 patients who were followed for 6 months revealed that neurological symptoms were reported in 19.7% of participants. Among the long-term neurological symptoms, anxiety (19%) and sleep disorder (18%) were the most prevalent symptoms followed by depression (16%), concentration difficulty (15%), and cognitive impairment (13%) [102]. Hartung et al. studied the frequency of cognitive impairment in 969 cases 6 to 11 months after acute COVID-19 infection. They reported that 26% of patients had mild cognitive impairment after 9 months of infection [103] (Fig. 4).

It is hypothesized that both central and peripheral disruptions of the KP are involved in the neurological manifestations seen in COVID-19 patients. Systemic inflammation, induced by SARS-CoV-2 infection, can alter KP metabolites in the periphery, which may then affect the CNS either through disrupted blood–brain barrier permeability or via signalling through peripheral nerves. Simultaneously, immune response within the CNS may lead to local dysregulation of the KP, contributing directly to neuroinflammation and pathology. Studies have shown alterations in the levels of KP metabolites, such as QUIN, and KYN, in patients with COVID-19, suggesting a link to neuroinflammatory processes [16]. For instance, the possible role of the KP in cognitive impairment associated with long COVID has been investigated by Cysique et al. They assessed mental health, cognition status, and KP metabolites in 128 post-acute COVID-19 patients. Their findings indicated that cognitive performance significantly declined in post-acute mild-moderate COVID-19 patients during the 12-month study period. They also found that cognitive impairment is significantly associated with KP metabolites such as QUIN, KYN and 3-HAA [16]. The KP dynamics showed decreased levels of KYN, KYN/Trp and anthranilic acid (AA) across the study period (2, 4, and 12 months post-COVID), whereas QUIN and 3-HAA had an increasing trend over time (2 to 8 months) [16].

The changes in enzymatic activity and metabolites of the KP in COVID-19 patients may indeed serve as important biomarkers for predicting neurological involvement. For instance, elevated levels of neurotoxic metabolites could indicate increased neuroinflammation and risk for developing neurological symptoms. These markers could potentially help in stratifying patients based on the risk of neurological complications, thereby guiding clinical management. While this area of research is promising, it is still in the early stages. The variability in timing, severity of infection, and individual patient factors, complicates the direct application of biomarkers as universal predictive tools. More longitudinal studies and larger patient cohorts are needed to validate the utility of KP metabolites as biomarkers for neurological manifestations of COVID-19.

#### The kynurenine pathway dysregulation and its implications for headache in long COVID

Headache has been identified as a common and significant issue in long COVID patients [104, 105]. Duration of headaches in long COVID patients can be prolonged, impacting their quality of life [106]. It has been suggested that long COVID headaches may develop due to the activation of an existing headache condition or in patients with history of migraines [104]. The studies have indicated that individuals experiencing prolonged headaches in long COVID reported frequent headaches during the acute phase as well with a considerable link between the severity of the headache in acute phase of COVID-19 and its extended persistence in long COVID [107, 108]. The pathophysiology of headaches in long COVID is complex and may involve sustained inflammation and hyperimmune activity. Several factors such as sleep disruptions, metabolic dysregulation of neurotransmitters like serotonin, dopamine, glutamate, and gamma-aminobutyric acid (GABA) could contribute to development of chronic headaches in long COVID [109].

The possible involvement of the KP in chronic headache in long COVID patients extends to its influence on glutamate receptors (*e.g.,* NMDA receptors) and excitotoxicity, which are relevant factors in headache and migraine pathophysiology [110]. NMDA receptors have been implicated in the pathophysiology of chronic headaches and migraines possibly through their role in central sensitization, a process that enhances the response of neurons to external stimuli [111]. Central sensitization in the trigeminovascular system, which is a key component in headache pathology, can lead to the lowered threshold for pain and the amplification of headache signals [112, 113]. The interaction between NMDA receptors and KP metabolites has been a subject of significant research interest due to its implications in various neurological diseases and psychiatric disorders. QUIN, a neurotoxic metabolite of the KP, is known as an NMDA receptor agonist, contributing to neurotoxicity. In contrast, KYNA acts as an antagonist at NMDA receptors, presenting a neuroprotective effect against excitotoxicity [114, 115]. It has been reported that levels of KYNA in serum of patients with chronic migraine significantly decreased by 25% compared to healthy controls [116]. The balance between QUIN’s agonistic action and KYNA’s antagonistic action on NMDA receptors is crucial in determining neuronal survival [117].

Beside the KP, alterations in serotonin system have been implicated in the pathophysiology of chronic headache and migraine [118, 119]. Trp can be metabolized into KYN and its derivatives through the KP, or serotonin, with the metabolism being influenced by Trp availability or immune system responses. Activation of IDO-1 during the infection and cytokine storm shifts the Trp metabolism toward the KP, causing lower availability of serotonin. Studies have reported that low levels of serotonin can lead to enhanced sensitivity in the trigeminovascular pathways associated with migraines [120, 121]. Moreover, positron emission tomography has revealed increased activity of serotonergic neurons in migraine patients, and selective serotonin receptor agonists like triptans have proven effective in migraine prevention and treatment [122].

#### Potential mechanisms linking the kynurenine pathway dysregulation to cognitive impairment in long COVID.

One of the potential mechanisms contributing to cognitive impairment in long COVID is the formation of amyloid fibrin microclots. The microclots induced by the spikes of SARS-CoV-2, a hallmark for both COVID [123, 124] and long COVID [125], reduce the flow of red blood cells to the capillaries and obstructing the transfer of oxygen leading to neuronal injury [126][127, 128]. The role of microclots in cognitive dysfunction extends beyond their direct impact. The microclots can initiate a cascade of secondary processes that further exacerbate cognitive declines. For example, amyloid fibrin microclots can exert pro-inflammatory activity and autoantibodies [127]. Moreover, it has been demonstrated that they can capture several inflammatory molecules with the ability to inhibit the breakdown of clots [125]. The involvement of the KP in the coagulation system has been previously reported [129, 130]. KP metabolites can contribute to thrombosis formation through the disruption of coagulation factor regulation in the plasma, causing endothelial cell dysfunction, and promoting overexpression of tissue factor (TF) in cells via the AhR pathway [131]. Metabolomics data have shown that two kynurenine metabolites, 5-hydroxy-N-formylkynurenine and N-formylkynurenine, are associated with venous thromboembolism [129, 132, 133].

An unbalanced microbiome may exacerbate systemic inflammation, potentially leading to neuroinflammation and subsequently impairing cognitive function. It has been reported that transferring an aged microbiome to germ-free mice induced age-associated phenotypes. Accordingly, decreased SCFAs producing bacteria in the gut are associated with cognitive decline [134]. Dysbiosis of the gut microbiota could be linked to long COVID symptoms. Gut microbiota dysbiosis including a decreased number of SCFAs-producing species was correlated with persistent symptoms in COVID-19 recovered patients for one-year follow-up [135]. Gut microbiota dysbiosis-induced inflammation is implicated in the dysregulation of the KP, a process increasingly recognized for its roles in neuropsychiatric disorders and systemic inflammation. This activation is thought to be mediated by pro-inflammatory cytokines, which are elevated in states of chronic inflammation and can induce the expression of IDO-1 [136, 137] leading to the synthesis of neuroactive metabolites which could be associated with cognitive impairment.

RNA viruses are known for inducing acute infections usually for a short duration followed by recovery and the development of immunity by the host. Nonetheless, the persistence of full or fragments of the virus in host tissues post-infection has been observed for some RNA viruses, which may result in ongoing or delayed infection complications [138]. The persistence of symptoms in long COVID suggests that viral remnants due to the delayed/inadequate clearance from monocytes, macrophages, and endothelial cells might play a role in the neurological symptoms of long COVID. For example, the gastrointestinal tract has been identified as a potential reservoir for SARS-CoV-2, with its nucleocapsid protein found in intestinal samples four months post-infection, even in mild cases [139]. Moreover, viral spike protein has been detected in blood, lung tissue, and circulating monocytes of patients, weeks to months after the original infection, and the persistence of these spikes is associated with long COVID symptoms [140–142]. Although the presence of viral fragments could have a beneficial role by facilitating the development and enhancement of the humoral immune system, this might also result in chronic activation of the innate immune system or mimicry of host antigens, which could trigger autoimmune responses [143].

Inflammation can be further increased by the reactivation of latent herpesvirus infections, especially Epstein-Bar virus (EBV) by COVID-19 during acute illness [144, 145]. This activation might be associated with long COVID symptoms including cough, fatigue, and cognitive impairments [146]. Although the EBV RNA has not been detected in long COVID cases, individuals suffering from long COVID had elevated levels of anti-EBV antibodies compared to those without long COVID symptoms [147, 148]. Importantly, EBV can induce IDO-1 activity through TNF-α- and IL-6-dependent mechanisms in monocyte-derived macrophages [149]. EBV reactivation and elevated levels of KP metabolites and inflammatory cytokines such as IL-6 could be linked to long COVID symptoms.

#### Involvement of the kynurenine pathway in long COVID-related depressive and PTSD symptoms

It has been hypothesized that COVID-19 patients with severe disease and overactivation of the immune system are at higher risk of depression after acute infection [150]. Mazza et al. (2020) studied psychiatric symptoms in 402 cases (265 male, mean age 58) surviving COVID-19 one-month follow-up after initial symptoms. Their results revealed that 31% of patients suffered from depression, 28% from post-traumatic stress disorder (PTSD), and 42% from anxiety in the post-illness period [150]. According to a meta-analysis study, the prevalence of depression was estimated at 26% among the general population during the COVID-19 pandemic [151]. In a virtual follow-up study (median, 65 days), 946 adults with the clinical diagnosis of COVID-19 including patients treated in the Emergency Department, inpatient wards, and intensive care screened for psychological morbidity. Of these, 80.3% (n = 760) of individuals scored more than 3/6 and 6/10 on the Patient Health Questionnaire 2-item scale for depression and Trauma Screening Questionnaire for PTSD and were consulted by a psychologist. Persistent physical and psychiatric symptoms were observed in 47% (n = 357) of patients. 10.5% (n = 80) and 13.5% (n = 105) of patients were determined positive for PTSD and depression, respectively. Interestingly, patients with the positive Patient Health Questionnaire 2-item scale and Trauma Screening Questionnaire were at significantly higher risk (p < 0.001) of experiencing persistent COVID-19 symptoms, particularly confusion, anorexia, myalgia, and breathlessness [152]. Severe COVID-19 patients present a concomitant significant increase in 1) IDO-1 activity, 2) increased production of KP metabolites, and 3) high levels of proinflammatory mediators including TNF-α and macrophage inflammatory protein-1 α (MIP-1α) both part of the cytokine storm [153, 154]. The chemokine MIP-1α which is mainly produced by macrophages and monocytes can be induced by PIC, a metabolite of the KP that regulates microglial inflammatory activity in the CNS. MIP-1α plays an important role in neuronal transmission and cognitive functions. Serum levels of MIP-1α are associated with depression severity [155]. The dysregulation of the KP has been reported by many teams as a key player in the development of depressive symptoms [156–158]. A recent study showed that depressive patients have elevated plasma levels of inflammatory mediators, especially TNF-α, which is associated with increased plasma KYN and KYN/Trp [159]. Therefore, IDO-1-mediated degradation of Trp through the KP can be associated with depression via two potential mechanisms: i) decreasing the bioavailability of circulating Trp as an essential amino acid for the synthesis of serotonin and ii) production of neurotoxic metabolites downstream of IDO-1 especially QUIN [160, 161]. It has been found that high concentrations of QUIN in blood and CSF are associated with major depressive disorder (MDD) and cytokine-induced depression [160, 162, 163]. Several mechanisms such as oxidative and nitrosative stress, serotonergic system dysfunction, inflammation, and formation of neuroactive metabolites *i.e*., QUIN could contribute to the development and progression of depressive symptoms [164–166].

#### The potential role of the kynurenine pathway in long-COVID-associated fatigue syndrome

According to recent reports, a significant population of COVID-19 patients has been suffering post-viral fatigue (PVFS) [167–169]. The results of a survey in the United States showed that 35% of 292 adults had not fully recovered 2–3 weeks after initial COVID-19 symptoms [167]. An Italian hospital reported that 87% of patients experienced ongoing fatigue 2 months after they had tested positive for COVID-19 [168]. As the clinical studies have progressed, the concept has been raised that SARS-CoV-2 can trigger a form of fatigue termed PVFS. It has been proposed that the symptoms of PVFS are highly similar to the Myalgic Encephalomyelitis Chronic Fatigue Syndrome (ME/CFS) and Fatigue Syndrome (FS)[170, 171], however, ME/CFS can be triggered either by infectious diseases such as mononucleosis or non-infectious causative agents. A recent study found that 67% of the adults who suffer from PVFS were female [169], like ME/CFS which has a gender bias toward females as well. A web-based survey including 3762 individuals reported that of those who suffered from PVFS, 80% were female. The most common symptoms have also been listed as fatigue, post-exertional malaise, and cognitive problems, which are the common symptoms of ME/CFS [172–175]. The underlying pathophysiology of both diseases is proposed to be chronic inflammation and activation of immune cells along with an elevation in proinflammatory mediators associated with clinical symptoms such as chronic fatigue and cognitive dysfunction. As mentioned earlier, increased proinflammatory mediators such as IFN-γ, TNF-α, and IL-6 can induce IDO-1 and increase the Trp catabolism through the KP leading to an increase in the production of neurotoxic metabolites (*i.e.,* QUIN). In addition to the neurotoxic effects of QUIN mentioned above, excessive levels of QUIN can negatively affect the NAD + metabolism [176, 177]. Trp catabolism via the KP is one of the three pathways for the de novo synthesis of NAD + . Under the inflammatory conditions, the synthesis of NAD + increases consistent with elevation in the synthesis of QUIN up to concentrations where saturation of the enzyme quinolinic acid phosphoribosyltransferase (QPRTase) (the enzyme responsible for conversion of QUIN to nicotinic acid mononucleotide) occurs. QUIN-induced oxidative stress is a driving force to activate the poly (ADP-ribose) polymerase-1 (PARP-1), an enzyme that is responsible for repairing damaged DNA under oxidative conditions [178]. Overactivation of PARP-1, which is a significant consumer of NAD + , leads to NAD + and ATP depletion that is associated with mitochondrial dysfunction and energy loss. Indeed, it has been shown that PARP isozymes are consistently upregulated by SARS-CoV-2 infection and their overactivation can depress the NAD metabolome [179].

## Conclusions

The acute phase of COVID-19 is marked by a significant alteration in the levels of KP metabolites, contributing to the cytokine storm and exacerbating the disease’s severity. These alterations are not only pivotal in understanding the acute infection’s pathogenesis but also in deciphering the lingering effects observed in long COVID syndrome. The sustained activation of the KP, associated with cognitive impairments and chronic fatigue, underscores the potential of KP as a therapeutic target and as a possible biomarker for long COVID in future studies.

Furthermore, the differential modulation of the KP in response to COVID-19, influenced by factors such as sex and severity of the disease, suggests a complex interaction between the virus, host immune response, and metabolic pathways. The evidence suggests that interventions targeting the KP could offer new avenues for mitigating the impact of COVID-19, particularly in managing the long-term sequelae. Future research should focus on elucidating the precise mechanisms through which SARS-CoV-2 influences the KP and exploring targeted interventions that could ameliorate both acute and chronic manifestations of COVID-19. Given the role of KP in immune modulation and neurobehavioral changes, targeting this pathway could help alleviate both acute and persistent symptoms associated with COVID-19. Potential strategies include the use of KP enzyme inhibitors to prevent the excessive production of neurotoxic metabolites and/or the administration of KP metabolites with neuroprotective properties. Clinical trials investigating these interventions could provide critical insights into their efficacy and safety in COVID-19 patients. The more comprehensive longitudinal studies should aim to track KP metabolite levels, immune response, and clinical outcomes in patients over time to elucidate the persistent effects of SARS-CoV-2 infection. Insights from longitudinal research could inform post-COVID-19 care, rehabilitation strategies, and the development of interventions to address long COVID symptoms. The integration of KP-focused research within broader COVID-19 studies will be crucial in uncovering the full extent of the virus’s impact on human health.

## Data Availability

No datasets were generated or analysed during the current study.
